# PSnpBind: a database of mutated binding site protein–ligand complexes constructed using a multithreaded virtual screening workflow

**DOI:** 10.1186/s13321-021-00573-5

**Published:** 2022-02-28

**Authors:** Ammar Ammar, Rachel Cavill, Chris Evelo, Egon Willighagen

**Affiliations:** 1 Department of Bioinformatics—BiGCaT, NUTRIM, Maastricht University, Maastricht, The Netherlands; 2 Department of Data Science and Knowledge Engineering, Maastricht University, Maastricht, The Netherlands

**Keywords:** Binding affinity, Mutation effect, Binding pocket, Virtual screening, AutoDock Vina, SNP, Database, REST API

## Abstract

A key concept in drug design is how natural variants, especially the ones occurring in the binding site of drug targets, affect the inter-individual drug response and efficacy by altering binding affinity. These effects have been studied on very limited and small datasets while, ideally, a large dataset of binding affinity changes due to binding site single-nucleotide polymorphisms (SNPs) is needed for evaluation. However, to the best of our knowledge, such a dataset does not exist. Thus, a reference dataset of ligands binding affinities to proteins with all their reported binding sites’ variants was constructed using a molecular docking approach. Having a large database of protein–ligand complexes covering a wide range of binding pocket mutations and a large small molecules’ landscape is of great importance for several types of studies. For example, developing machine learning algorithms to predict protein–ligand affinity or a SNP effect on it requires an extensive amount of data. In this work, we present PSnpBind: A large database of 0.6 million mutated binding site protein–ligand complexes constructed using a multithreaded virtual screening workflow. It provides a web interface to explore and visualize the protein–ligand complexes and a REST API to programmatically access the different aspects of the database contents. PSnpBind is open source and freely available at https://psnpbind.org.

## Introduction

Over the last 50 years, pharmacogenomics has studied the genetic basis for inter-individual drug response variability [1]. Many factors are involved in patient-drug response, for instance, environmental and behavioral factors. At the same time, genetic factors also play an essential role [2]. Genetic factors that can have functionally substantial consequences on drug response are numerous. For example, they include genetic variants’ effects on the protein structure and stability, DNA transcription, and mRNA regulation [1]. Studies have shown that 80% of patients carry at least one functional variant in the drug targets of the top 100 most commonly prescribed drugs in the United States [3]. The variation in drug-response at the protein level and its underlying mechanisms are of a significant interest in developing new drugs with an estimate of six single nucleotide polymorphisms (SNPs) affecting five different FDA-approved drugs carried by every individual [4].

Mutations that occur in the binding site of a target protein may change the protein–ligand binding affinity, which can lead to a substantially different phenotype resembling lower efficiency of the drug or higher off-target binding affinity that could lead to side effects [5]. Nevertheless, large-scale studies of the effect of SNPs occurring at the binding site of proteins on a structural level do not exist to the best of our knowledge. Besides, studies often focus on one protein with a limited number of variants [6–13]. For example, Doss et al. studied the effects of SNPs in the anaplastic lymphoma kinase (ALK) protein on the patient’s drug response including 21 binding site related SNPs. Furthermore, these studies need substantial computational power because they mostly rely on demanding molecular dynamics simulations. As a result, performing such studies on a large-scale, including a wide range of proteins and ligands, is expensive and laborious.

Several studies [14–23] tackled the topic of creating a database or web server for mapping SNPs onto protein structures, as shown in Table 1. However, we identified several issues after a thorough analysis of these resources. For example, none of these projects provide data about the binding site residues’ mutations or their effect on ligand binding. Besides, most of them [15, 16, 18–20, 22] are either no longer available or outdated. Others [14, 17, 21, 23] are not downloadable and do not provide application programming interfaces (APIs) for programmatic access. Having a large database of protein-ligand complexes covering a wide range of binding pocket mutations and a large small molecules’ landscape is of great importance for several types of studies. For example, developing machine learning algorithms to predict protein–ligand affinity or a SNP effect on it requires an extensive amount of data with a wide coverage of mutation types and small molecules. Also, studies of protein–ligand interactions and conformer orientation changes across different mutated versions of a protein require such a database.

For those reasons, we decided to develop the PSnpBind database (https://psnpbind.org) to address the lack of large datasets about the binding affinity of binding site mutated protein-ligand complexes and to provide a base for new studies in related fields like drug discovery, pharmacogenomics, and structural bioinformatics.

**Table 1:** List of databases related to SNPs effect analysis and visualization

**Database**	**Last update**	**Comment**
MSV3d [14]	2016, Not downloadable, (web only)	Mutated structures built using MODELLER. The website also contains conservation and physio-chemical changes. SwissVar and dbSNP are the main sources of SNPs
PinSnps [15]	2013	Exploring the impact of SNPs on Protein Domains and Complexes
LS-SNP/PDB [16]	2009, Not available anymore	
G23D [17]	2016, Not downloadable, (web only)	Used software: SCCCOMP and SCWRL for Side chain modeling, JSmol for molecular graphics, I-mutant and FoldX for thermostability prediction
SNPs3D [18]	2008, Not downloadable	SNP impact on protein structure and function. A Support Vector Machine (SVM) model was used to find the separation pattern between a set of diseases and non-deleterious SNPs. The resulting pattern was then validated using a different set of diseases and non-deleterious SNPs.
SAAPdb [19]	No longer maintained	A newer project SAAPdap/SAAPpred is available - analysis pipeline for examining the structural effects of mutations/prediction of pathogenicity.
SNP2Structure [20]	Not available anymore	
PhyreRisk [24]	2019, Not downloadable (web only)	A dynamic web application to bridge genomics, proteomics and 3D structural data to guide interpretation of human genetic variants.
toposnp [21]	2019, Databases are up to date, Not downloadable, (web only)	Topographic mapping of Single Nucleotide Polymorphism
coliSNP [22]	Not available anymore	
StructMAn [23]	2016, Not downloadable (web only)	Annotation of non-synonymous single-nucleotide polymorphisms (nsSNPs) in the context of the structural neighbourhood of the resulting amino acid variations in the protein.

## Construction and content

**Fig. 1:**
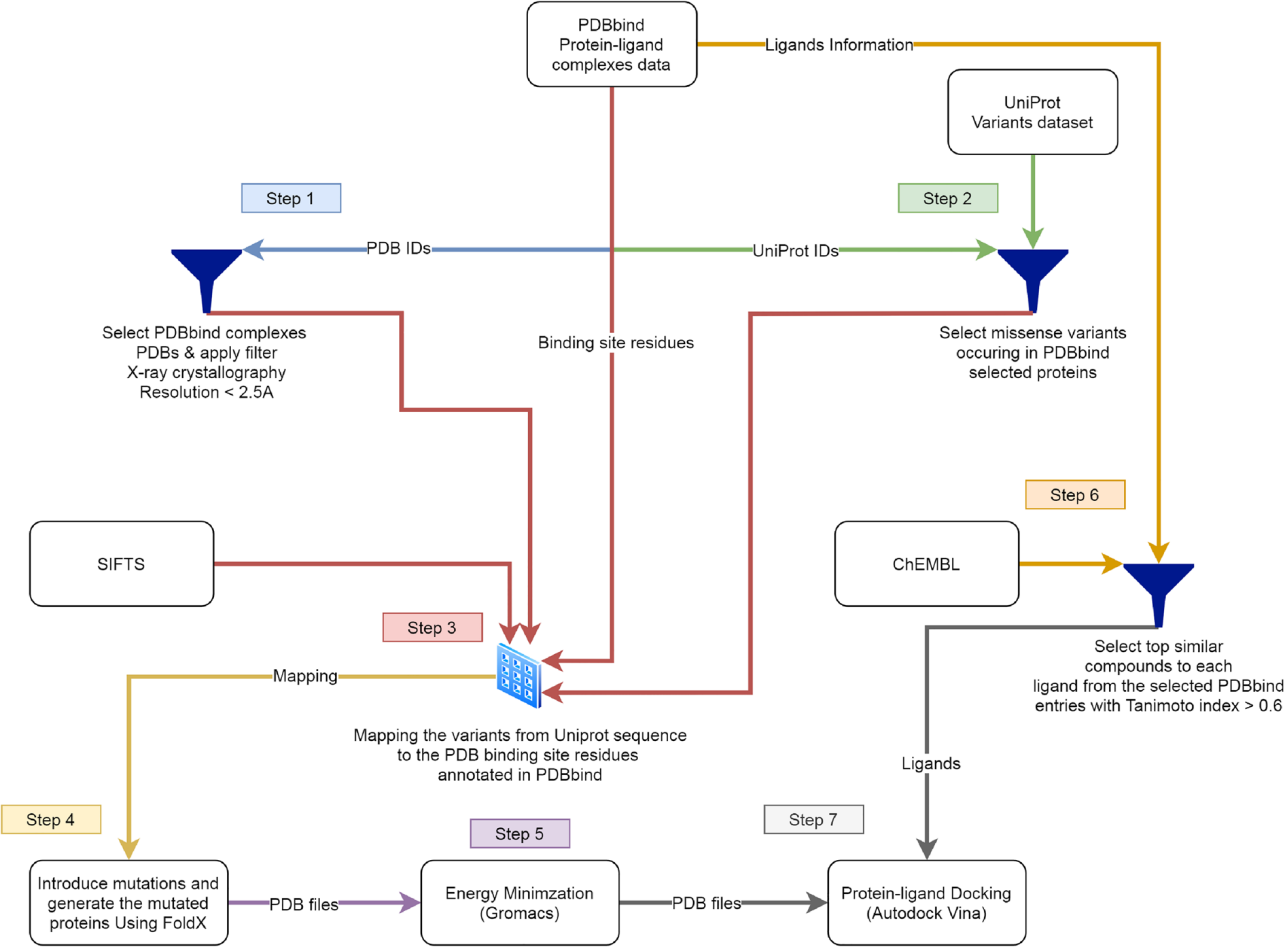
Methodology workflow. Steps 1, 2 and 3 filter the data from the main sources and map them together. Step 4 and 5 prepare the selected protein PDBs and their mutated versions for docking. Step 6 prepares the ligands. Step 7 performs the docking

Figure 1 shows the methodology of PSnpBind database construction. The methodology is composed of 7 steps targeting three main processes: protein structures preparation, ligand structures preparation and protein–ligand docking. The following subsections explains in detail each step of the methodology.

### Data sources

Several data sources were used to integrate the information about proteins (structures, sequences, and variants), ligands structures and protein-ligand complexes structures and relevant information. Figure 1 shows the main data sources used and the filtering criteria applied on each one.

#### PDBbind

PDBbind [25] provides a comprehensive collection of the experimentally measured binding affinity data for all types of biomolecular complexes deposited in the Protein Data Bank [26]. The entire PDB was screened to identify complexes and build the PDBbind database. This data source provides the biomolecular complexes grouped into four groups (protein–ligand, protein-nucleic acid, nucleic acid-ligand, and protein-protein complexes). For our project, we were only interested in protein-ligand complexes which are also distributed over three datasets:

- General set: all protein–ligand complexes from PDB.
- Refined set: a standard data set for docking and scoring studies.
- Core set: includes high-resolution crystal structures and reliable binding constants.

Building the PDBbind database included collecting all the complexes from PDB without performing structural optimization or any type of transformation on the coordinates. Binding affinity data (dissociation constant (K_d_), inhibition constant (K_i_), and concentration at 50% inhibition (IC_50_)) were collected from the primary references of the deposited entries. The authors applied a priority order of K_d_ > K_i_ > IC_50_ when more than one value appeared in the references, and they only recorded the data with the highest priority. Also, in case of binding affinities measured under different conditions (temperature and pH) for any complex, only the results measured at neutral pH and room temperature or in assay conditions close to that were recorded.

PDBbind also provides a residue-level annotation for the amino acids involved in the binding pockets of the protein-ligand complexes. It is a crucial piece of information for our project to map the missense SNPs onto the binding pocket residues.

We chose to use the PDBbind core set version 2016 (also called Comparative Assessment of Scoring Functions “CASF” dataset) in this work mainly because it is a concise version of the refined set where all redundancy is eliminated. Besides, the size of the dataset is smaller and more practical to work with. CASF 2016 was downloaded on October 6th, 2019, from the PDBbind website, and contained 285 high-quality protein–ligand complexes, out of which 123 complexes are for human proteins.

#### UniProt

UniProt [27] was used as a source for protein sequences and human protein variants. The human variants dataset from UniProt contained the amino acid substitutions resulting from missense SNPs in the protein-coding regions in the human genome. UniProt provides manually reviewed protein-altering natural variants imported from the publicly accessible variant resources such as Ensembl Variation [28] and ClinVar [29] databases. Four types of variants are included in this dataset annotated with Sequence Ontology (SO) identifiers: missense variants (SO:0001582), stop lost (SO:0001578), stop gained (SO:0001587), and initiator codon variants (SO:0001582). The version released on October 16th, 2019 was used and only the missense variants were considered. The used version contains about 29 million variants occurring in about 19000 human genes.

#### SIFTS

SIFTS (Structure Integration with Function, Taxonomy, and Sequence) is a project in the PDBe-KB resource for residue-level mapping between UniProt and PDB entries [30]. It is a close collaboration between the Protein Data Bank in Europe (PDBe) and UniProt. It uses NCBI taxonomic identifiers as a standard way of representing taxonomic information for all PDB entries within the PDBe database. For all the protein sequences in the PDB that are present in the UniProt database, cross-references to UniProtKB are added in SIFTS. SIFTS provides an accurate mapping of the sequences from PDB entries on to corresponding UniProt entries. It also contains a mapping to both:

- SEQRES record in the PDB entry: the complete sequence of the protein used in the experiment.
- ATOM record in the PDB entry: the observed residues in the crystal structure.

This dataset is a vital resource in this project in order to be able to map the protein-altering variants in the protein sequence to the binding pocket residues in the crystal structure.

#### ChEMBL

ChEMBL is an open database that contains functional, binding, and ADMET information for many drug-like bio-active compounds (about two million compounds) [31]. It is maintained by the European Bioinformatics Institute (EBI), of the European Molecular Biology Laboratory (EMBL), based at the Wellcome Trust Genome Campus, Hinxton, UK. The data in ChEMBL is manually curated from thousands of publications and dozens of deposited datasets. The need for a chemical compounds dataset is to create a chemical library from similar compounds to the ones selected from PDBbind.

The created library was used to perform molecular docking against the proteins selected from PDBbind with their mutated version to obtain a reference dataset large enough to train a machine learning model on it. ChEMBL version 25, released on February 1st, 2019, was used in this work.

### Dataset construction

To build the dataset of mutated protein-ligand complexes, we needed to identify the natural mutations occurring in binding pockets of human proteins selected from CASF. The next sections describe the process in detail.

#### Steps 1, 2, and 3: obtaining the list of CASF human proteins binding pockets missense variants

First, the PDBbind dataset was downloaded and filtered to include only high-resolution structures obtained with X-ray crystallography (resolution $\le$ 2.5 Å). Even though PDBbind contains structures obtained by both X-ray and NMR, we only selected the X-ray based structures as the number of NMR structures is tiny compared to X-ray. There are differences between the two approaches with effects on the obtained structures like the number of inter-residue contacts and the main-chain hydrogen bonds [32]. These differences will, therefore, require performing different kinds of preprocessing and analysis, and since the number of NMR structures is low, we decided to ignore them.

In X-ray crystallography, heavy atoms scatter the X-rays resulting in a diffraction pattern. This pattern can be computationally converted back to a detailed protein structure. Resolution is a measure of the level of detail present in the diffraction pattern. A threshold of 2.5 Å for structure resolution was used [33, 34] to ensure that atomic details can be seen, while in structures with low resolution, only the basic contours of the protein chain can be seen.

The entries of PDBbind were also filtered to include only the ones with a UniProt ID. The dataset included multiple complex structures for the same UniProt proteins (i.e., proteins complexed with more than one ligand). Therefore, entries were aggregated by UniProt ID, and the proteins with the highest resolution were selected from each group. Each ligand that shares a complex with those proteins was also stored.

Next, the variant data from UniProt was downloaded and filtered to only include missense variants. Then, by doing an “inner join” over the UniProt ID column between the UniProt variants and the PDBbind entries selected from the previous step, only those missense variants occurring in the proteins of the PDBbind entries list were selected. All the duplicates were removed, and the joining resulted in 11749 missense variants belonging to 26 proteins (26 unique UniProt IDs). Since the variants dataset only contains human variants, the resulting dataset only contained human proteins with their variants. Table 2 shows an example list of filtered variants from the UniProt variant dataset.

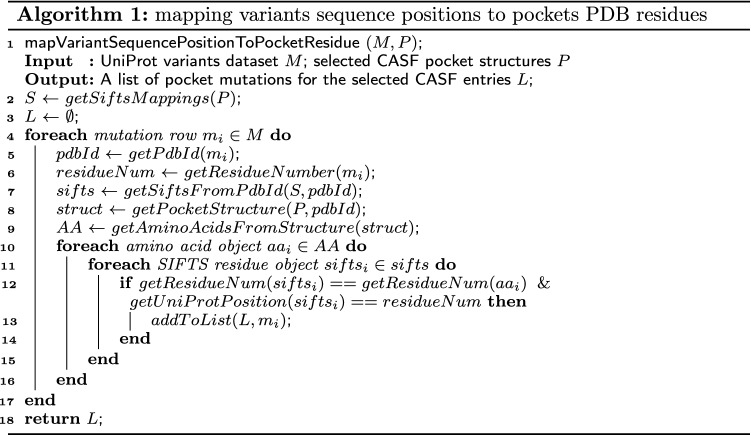



After that, the SIFTS mappings for the 26 human proteins were downloaded from the PDB website. Next, the BioJava [35] library was used to parse the SIFTS mappings. In conjunction with the binding pocket annotations (binding pockets residue numbers) of the PDBbind entries, only those variants occurring in the binding pocket were retained. All the duplicates were removed, and the joining resulted in 705 missense variants belonging to 26 protein binding pockets (26 unique UniProt IDs). Algorithm 1 shows pseudocode for the matching between variants sequence location and pocket residues in the PDB structure.

**Table 2:** Example list of CASF human proteins variants selected from the UniProt variants dataset

**Uniprot ID**	**Missense SNP**	**PDB ID**	**Source AA**	**Target AA**	**Residue Num.**	**Chain**
O14757	p.Leu92Phe	3jvr	L	F	92	A
O14757	p.Phe93Val	3jvr	F	V	93	A
O14757	p.Ile96Val	3jvr	I	V	96	A
O14757	p.Gly101Cys	3jvr	G	C	101	A
O14965	p.Gly140Ala	3up2	G	A	140	A

**Fig. 2:**
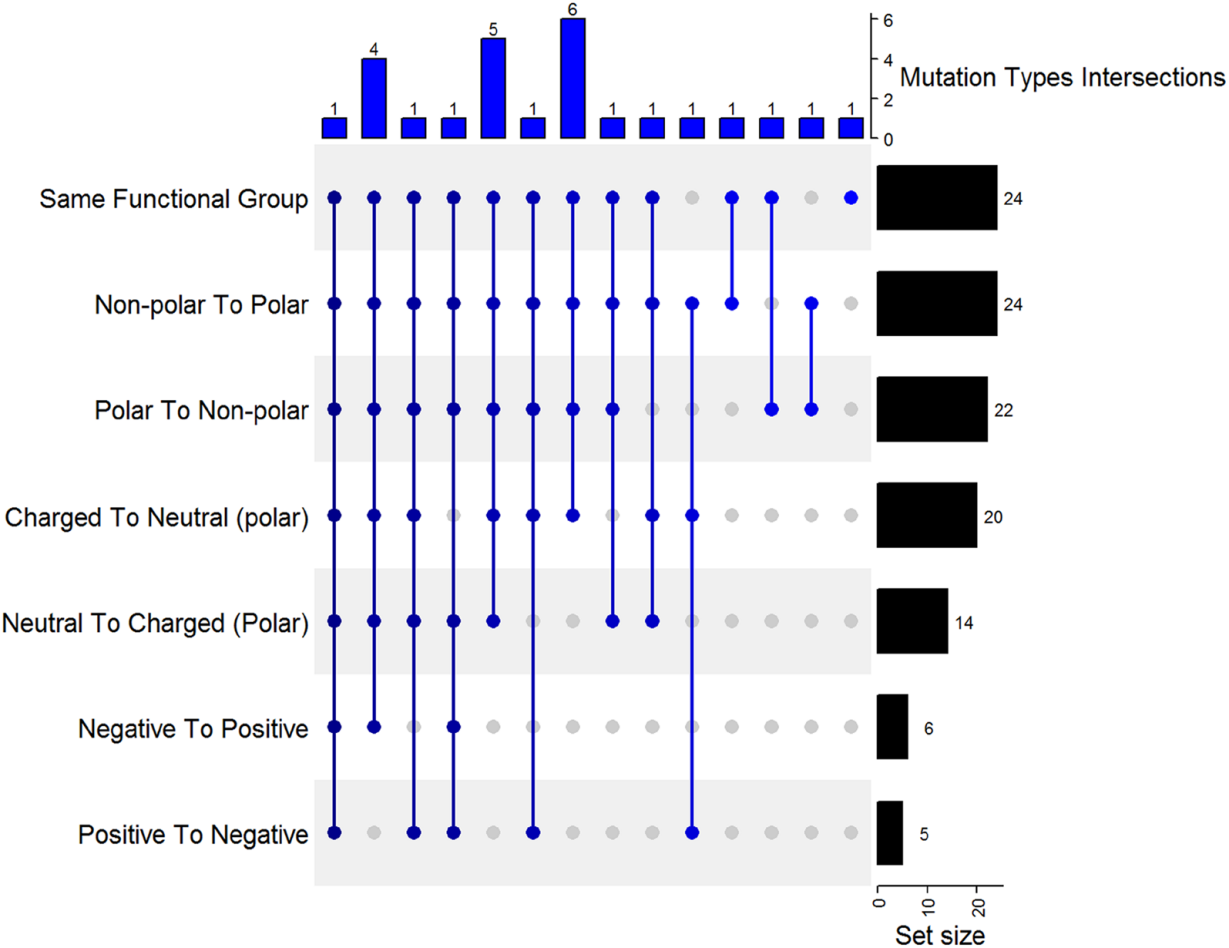
UpSet plot showing the availability of mutation types across the selected PSnpBind proteins. X-axis shows the number of proteins having the corresponding intersection between the mutation types. Y-axis shows the number of proteins having each mutation type

The residues where the mutations occurred were further analyzed to understand their nature by assigning the wildtype and the mutation amino acids in each residue location to one of the seven functional categories (polar, non-polar, neutral polar, charged polar, negatively charged, positively charged). Next, an UpSet plot was generated to quantify the frequency of the mutation types (transition from one group to another) across the 26 proteins in the dataset. The UpSet plot was generated using the ComplexHeatmap R package [36] as shown in Fig. 2.

Figure 2 shows, on the X-axis, the mutation types intersections reported in the obtained dataset (steps 1,2 and 3). For example, on the far left, there is one protein having binding pocket mutations from all the seven mutation types. Similarly, on the far right, there is one protein with only one type of mutation (same functional group, in this case). Also, a third example from the middle, there are 6 proteins having mutations belonging to four types (same functional group, polar to non-polar, non-polar to polar and charged to neutral). On the other hand, on the Y-axis, it shows the number of proteins having a certain type of mutation. For example, on top, there are 24 proteins having mutations from the “Same functional group” type. Another example, the last row, there are five proteins having mutations of the type “positive to negative”.

#### Step 4: Introducing the missense variants to the protein structures

After finding the missense variants in the binding pockets of the selected proteins, mutations were introduced to the protein structures using FoldX version 5.0 [37], one of the best stability predictors upon mutation [38, 39]. In this process, the targeted amino acid needs to be replaced with the mutated amino acid, and a proper side chain must be determined. Also, the structure needs to be optimized to incorporate the physio-chemical changes resulting from substituting the amino acid, and FoldX takes care of modeling these changes. FoldX is an empirical force field developed to evaluate the effect of mutations on the folding, stability, and dynamics of proteins and nucleic acids in a fast manner. FoldX identifies the most likely mutant residue side-chain rotamers taking into consideration the laws governing preserved angle conservation. FoldX utilizes a linear combination of empirical terms to calculate the energy fold change upon inducing the mutation, representing the effect of mutations on the protein structure in kcal/mol as described in its main work [37].

The FoldX “BuildModel” command takes the PDB structure file as input besides a text file named “individual_list.txt” containing the mutations wanted to be introduced to the structure separated by a comma. The syntax of representing the mutation in FoldX is (WT residue, chain, residue number, mutant residue). In our case, we only need to introduce a single mutation at a time, so we do not have comma-separated values. For example, using the variant information from Table 2, the mutations list would be as follows:

- LA92F;
- FA93V;
- IA96V;
- GA101C;
- GA101S;
- RA137C;
- RA137G;
- RA137H;
- GA140A;

#### Step 5: Energy minimization for the protein structures

The next step is energy minimization (EM) on the mutated protein structures. Gromacs version 2019.3 [40] with CHARMM27 force field (which is CHARMM22 that is revised to include CMAP corrections and bundled with the CHARMM program version 27) [41] and the TIP3P water model [42] were used to remove bad contacts, hindrance-causing torsion angles, etc.

The protein structures were solvated in a cubic box of TIP3P water molecules at a distance of 1.2 nm (12 Å) from the solvent. The system’s net charge was neutralized by adding enough ions in correspondence to the type and amount of the protein charge. A cutoff of 1.2 nm for both short-range van der Waals and electrostatic interactions was used, and PME (Particle Mesh Ewald) was used for long-range interactions in all minimizations.

For structures that contained ions in the binding site, those ions were added and position restraints were applied to the protein structure. Metal ions play important roles in biological processes like respiration and the structural stability of protein folds [43]. For example, one or two zinc ions (Zn^2+^) can be coordinated in a small protein structural motif, called “zinc finger,” which can be found in protein binding pockets in order to stabilize the fold.

Energy minimization was performed using the steepest descent algorithm. A maximum force of 100 kJ/mol/nm on any atom of the system was set as the end goal for convergence. A maximum number of 50000 steps of minimization was used. After EM, a potential energy plot was generated for each simulation to examine how the minimization went.

**Fig. 3:**
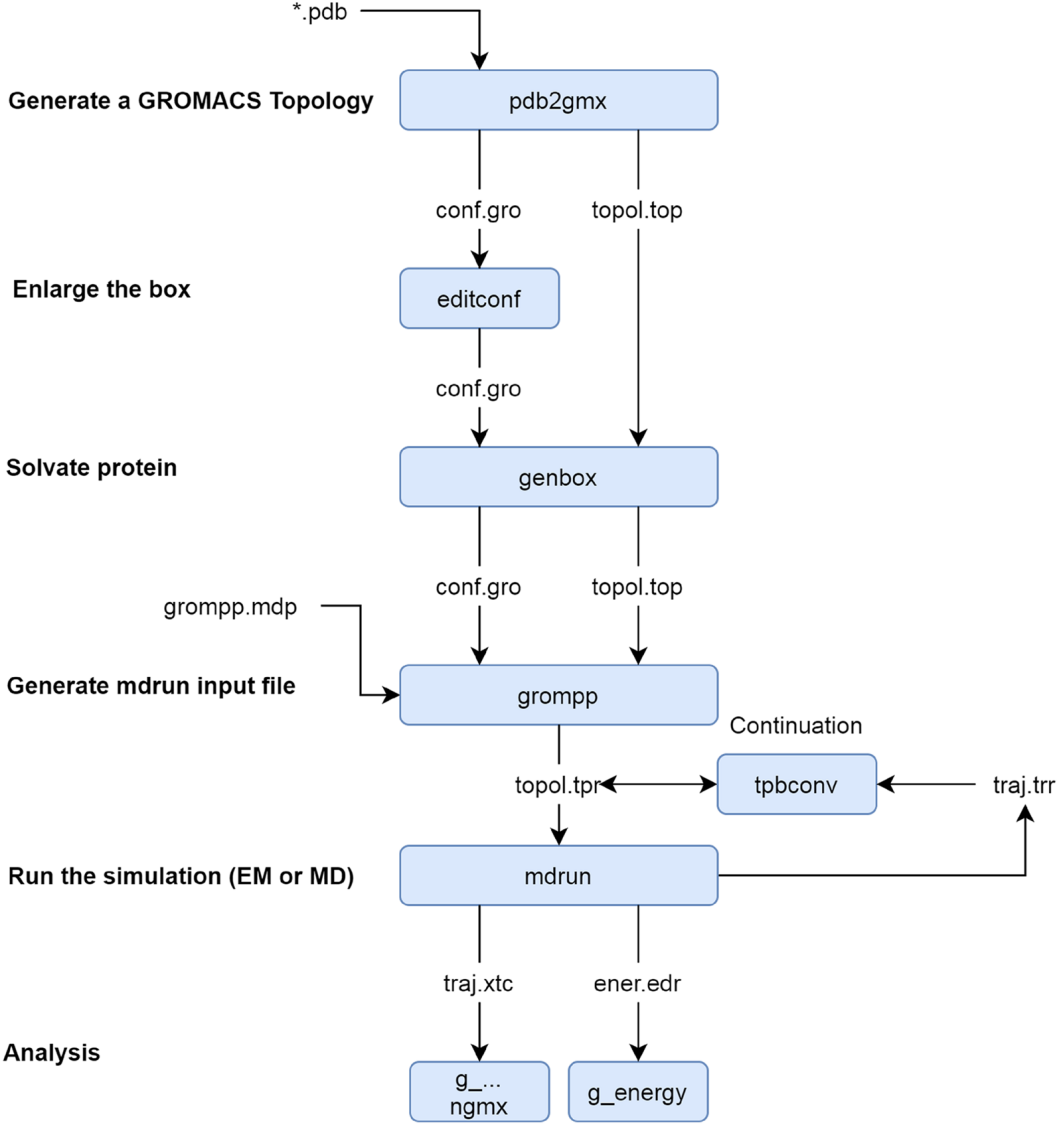
Gromacs energy minimization flowchart

Lastly, simulation trajectories were exported to the final PDB files using the “trjconv” program in Gromacs. Figure 3 shows a flowchart of Gromacs EM and MD simulations redrawn from [44]. Gromacs energy minimization protocol was followed from [44, 45], and the Gromacs manual (http://manual.gromacs.org/documentation/2019/manual-2019.pdf), and several settings and configurations were examined from the literature [46–48].

#### Step 6: Obtaining similar ligands for docking

A library of chemical compounds needs to be created to carry out a docking experiment against the mutated proteins. This library needs to be large enough to build a reference dataset of mutated protein–ligand binding affinities. Since the set of ligands binding to the selected proteins from PDBbind is too small to train a robust machine learning model that covers a wide range of ligands, such a library is needed.

ChEMBL [31] was chosen as a source for chemical compounds because it only contains bio-active compounds, and that aligns with the protein-ligand docking use case, the aim of the work. OpenBabel toolbox version 2.3.2 was used to prepare the chemical compounds sets [49] as in the following paragraphs. The ChEMBL dataset was downloaded in structure-data file format (SDF), and OpenBabel was used to create a fast search index. The index is a new file that stores a database of fingerprints for the molecules indexed. However, the index will allow significantly faster searching and similarity comparisons. The default fingerprint in OpenBabel was used to perform similarity search which is FP2, a path-based fingerprint which indexes small molecule fragments based on linear segments of up to 7 atoms.

Next, for each group of ligands belonging to a selected PDBbind entry, a similarity search was performed against ChEMBL to select similar compounds to each one of them. A similarity threshold (Tanimoto index) of 0.6 was chosen. The rationale behind choosing a low threshold is the need for compounds with a low similarity that will probably result in a low binding affinity. Including low binding affinities helps to cover a wider distribution of binding affinities which can be used to train machine learning models for binding affinity prediction applications. The Tanimoto index is a popular similarity coefficient used to measure the similarity between pairs of molecules [50]. The Tanimoto Similarity Coefficient is a generalization of Jaccard similarity, which is applicable only for binary data. In the case of OpenBabel, Tanimoto similarity is applied to fingerprints generated for molecules as vectors of binary values. The Tanimoto coefficient takes values between 0 and 1 (where 1 is the highest similarity).

Next, the similar molecules collected as a single file were split into one file per molecule, and converted to mol2 format needed for molecular docking. After that, the resulting molecules were energy minimized with OpenBabel using the MMFF94 force field [51]. The energy minimization of the ligands was performed using the steepest descent algorithm with a maximum step count of 2500. The molecules that failed the minimization were excluded from docking experiments.

#### Step 7: protein–ligand docking using AutoDock Vina

Modulating the function of proteins by small molecules has been an active research area with applications in drug design and development. To quantify the binding of a ligand to its target protein, a commonly used measure is their binding affinity, which describes how strongly the ligand binds to its biological counterpart. Binding strength can be measured experimentally by Microscale thermophoresis (MST, labelled and unlabeled proteins) [52, 53], Nuclear Magnetic Resonance techniques (NMR) [54–56], Isothermal Titration Calorimetry (ITC), Surface Plasmon Resonance (SPR), and Fluorescence Polarization (FP) methods [57]. Computational methods for the calculation of binding affinity range from rough estimates as in molecular docking, to more rigorous force fields in molecular dynamics (MD) simulations and Quantum Mechanical (QM) calculation [57].

Predicting interactions between ligands and proteins is a crucial element in the drug discovery process [57, 58]. In order to perform a quick search for molecules that may bind to targets of biological interest, computational techniques such as structure-based drug designing (SBDD) are carried out. SBDD includes structure-based virtual screening (SBVS) or molecular docking, followed by Molecular Dynamics [58]. Due to its ability to predict the ligand-binding affinity and conformation inside the receptor binding site with high accuracy, molecular docking is one of the most frequently used methods in SBDD [58]. A thorough search in three-dimensional spaces is performed by docking methods to find probable interactions, and a scoring function is used to rank the candidates correctly.

The molecular docking was performed using AutoDock Vina software [59]. AutoDock Vina was developed with ease of use in mind. It fulfills the need of a full-stack docking method that requires no expert knowledge to perform. It is freely available and uses well-tested default methods to perform highly optimized docking experiments. AutoDock Vina provides the binding affintiy as an approximation of the change in Gibbs free energy ($\Delta G$ or delta G), which is a negative number when the protein-ligand system reaches an equilibrium state.The magnitude of the negative $\Delta G$ determines the protein–ligand stability, or alternatively, the binding affinity. AutoDock Vina computationally calculate the $\Delta G$ using a scoring function to obtain the lowest-scoring conformation and reports the binding affinity using kcal/mol as the unit of binding affinity. The docking requires two main inputs, coordinates for receptor and ligand, to find the ideal docking poses. Receptor coordinates can be obtained from crystallography or NMR spectroscopy, while ligand coordinates are usually generated from SMILES [60] (Simplified Molecular-Input Line-Entry System) strings.

The docking protocol was followed from [61]. First, the protein structures as PDB files were prepared using AutoDockTools 1.5.6 (ADT). In this step, atom coordinates were parsed, and “autodock type” was assigned to each atom by ADT. Next, all hydrogens were added, non-polar hydrogens were merged, and the formatted receptor was written to a ’pdbqt’ file. A similar process was performed for the ligands, which were provided as mol2 files. Ligands files were parsed, and atom types were assigned to each atom. Next, all hydrogens were added, non-polar hydrogens were merged, rotatable bonds were defined, and the formatted ligands were written to a ’pdbqt’ file. Autodock Vina still needs a configuration file besides the receptor and ligand files.

The configuration file contains coordinates and dimensions of the grid box where the docking will take place. The grid box, when the binding pocket is known, should encapsulate the binding pocket and cover the entire cavity to where the ligand may bind. Defining the grid box was implemented programmatically by reading the coordinates of the binding pocket residues (since the pocket is annotated in PDBbind). Next, the coordinates of the grid box corners were defined to include all the atoms of all pocket residues. The center of the search space was set to be the center of the grid box. None of the grid boxes exceeded 27000 Å^3^ of volume, which is the recommended threshold by Autodock Vina, where large search spaces increase the docking time and require a more exhaustive search. The number of poses to be returned was set to 3, and the search exhaustiveness was set to 12 (default 8) to ensure better coverage of the search space and the binding poses. The docking experiment’s implementation used both internal and external parallelization by utilizing multiple cores (12 cores) for the single docking experiment provided by the “cpu” configuration parameter, and running multiple dockings in parallel at the time as recommended by an extensive study on AutoDock Vina in [62].

Since Vina uses a stochastic search method, random numbers are used in the process. Therefore, a seed was used to allow reproducibility. Also, the same seed was used for all performed docking experiments. The same parameters were also unified to ensure maximum reproducibility on the computing platform used for conducting the experiment. To test if the seed has an effect on the docking results, 10 random seeds were tested by performing the docking on the 26 selected protein–ligand CASF entries and calculating the correlation with the experimental values for each docking set. The evaluation didn’t result a considerable change in the docking behaviour. The dockings were performed using ten nodes in the data science research infrastructure provided by our institution (DSRI, https://maastrichtu-ids.github.io/dsri-documentation/) (128 CPUs each). The time required for each docking was recorded along with CPU usage. Table 3 shows a summary of the proteins, mutations, ligand, and dockings performed to obtain a reference data set for binding affinities. The docking experiments result logs were parsed and aggregated for further analysis of the docking performance.

**Table 3:** Summary of selected protein structures, mutations, ChEMBL selected ligands, and the number of dockings ordered by the PDB ID

**UniProt ID**	**PDB ID**	# ** of protein structures**	# **of ligands**	# ** of dockings**
P00749	1owh	38	1901	72225
P11309	2c3i	18	1240	22316
P18031	2hb1	18	419	7531
P03372	2pog	13	7017	91214
P00918	2weg	22	1013	22281
P00742	2y5h	33	667	22010
P07900	3b27	21	1954	41023
P10275	3b5r	83	466	38671
P39086	3fv1	43	345	14782
O14757	3jvr	11	631	6933
P24941	3pxf	10	505	5044
P37231	3u9q	20	606	12114
P56817	3udh	5	2127	10635
O14965	3up2	18	895	16109
P00734	3utu	27	1796	48492
P03951	4crc	50	690	34496
Q16539	4dli	9	1320	11878
P23458	4e5w	9	1090	9801
P39900	4gr0	38	1090	41419
Q9H2K2	4j21	17	3295	56001
O60674	4jia	7	848	5930
Q08881	4m0y	19	169	3197
P00519	4twp	26	795	20662
O60885	4wiv	3	917	2747
P04637	5a7b	160	113	17996
Q9Y233	5c28	13	352	4567
	Total	731	32261	640074

**Fig. 4:**
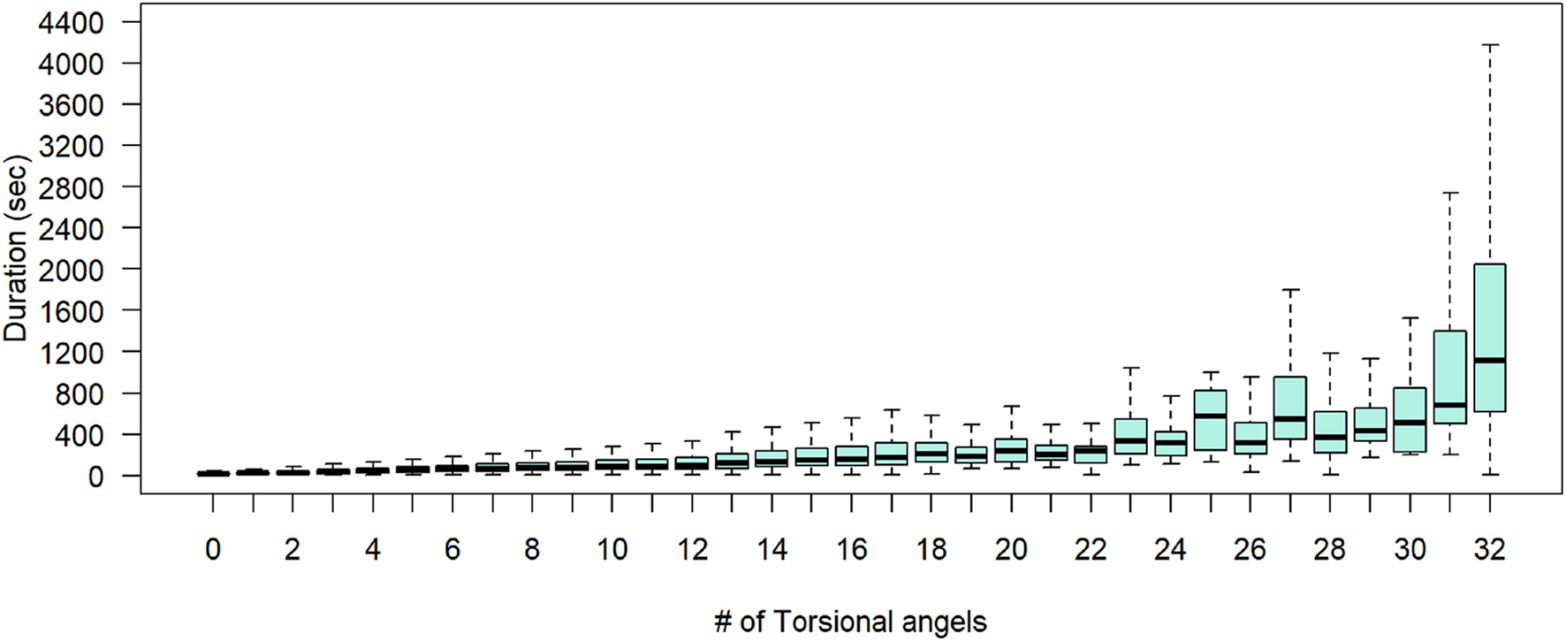
Docking performance—Duration versus number of torsion angles

**Fig. 5:**
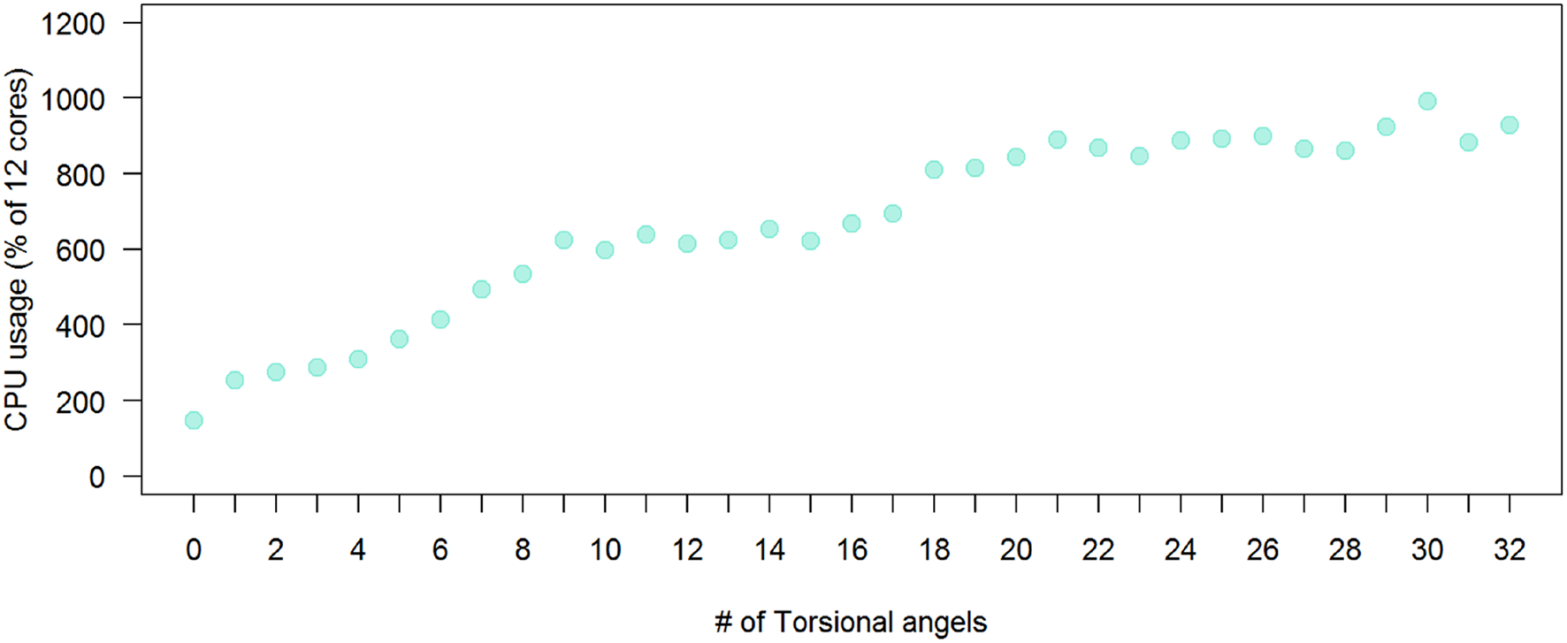
Docking performance—CPU usage versus number of torsion angles

The duration of each docking experiment and the CPU usage was recorded. Twelve cores were allocated for each docking, so the CPU usage value is a percentage of 1200%. The CPU usage and docking durations were grouped by the number of torsion angles of the ligands, respectively, and the median was calculated for each group. Figure 4 shows the relation between the number of torsion angles and the docking duration. Similarly, Fig. 5 shows the relation between the number of torsion angles and CPU usage. A linear relation was observed between the number of torsion angles and CPU usage. Both figures clearly show a direct relationship where the increase in the number of torsion angles leads to an increase in both docking duration and CPU usage. These results were expected because AutoDock Vina generates conformations by rotating each rotatable bond by a certain interval. For example, if a ligand has one torsion angle and Vina samples conformations at 10Å interval, then the number of possible conformations is $360/10 = 36$. In another example where the ligand has five torsion angles, the number of generated conformations would be $(360/10)^5= 60466176$ conformations. The number of computations is proportional to the number of torsional angles, which is reflected in a longer duration and higher CPU usage. AutoDock Vina allows no more than 32 torsion angles, where a larger number of torsion angles leads to impractical time-wise calculations.

The median duration of the total docking experiments is 57.54 seconds, with a 1st quartile duration of 32.74 seconds and a 3rd quartile duration of 97.19 seconds. The median CPU usage of the total docking experiments is 398% ($\sim$4 out of 12 cores) with a 1st quartile CPU usage of 213% ($\sim$2 out of 12 cores) and a 3rd quartile CPU usage of 699% ($\sim$7 out of 12 cores). The CPU usage results show that the docking performance could be optimized better by allocating a smaller number of cores per docking (7 cores, for example), which allows more dockings to be performed in parallel.

### Dataset construction implementation

All tools and packages used and developed for this project were encapsulated in independent Docker containers. The Docker containers were deployed on a cluster infrastructure running OpenShift, a container-orchestration system based on Kubernetes [63]. The code and the Docker images used in this project can be found in this GitHub repository: https://github.com/BiGCAT-UM/PSnpBind-Build. The GitHub repository describes the individual steps for building the PSnpBind database where each tool involved has its own repository. The GitHub repositories are linked to DockerHub, a cloud-based repository for automatic building, storing and distributing container images. DockerHub automatically rebuilds the Docker image after each commit to the corresponding GitHub repository. Hence, it saves time and effort. For deployment, the DSRI available at Maastricht University was used to deploy the dockerized tools used in this research. DSRI through OpenShift platform can deploy Docker images by grabbing them from DockerHub which also facilitates the integration between the source projects on GitHub and the production deployments. Ten nodes from DSRI were utilized to perform the calculations in the different steps of the methodology. Each node provides 128 cores to perform computations in parallel. That adds up to 1280 cores that were used to perform the most computationally-extensive steps: energy minimization and docking. Finally, all the repositories where preserved through Zenodo [64] and a DOI was minted for each one of them.

### Web application implementation

**Fig. 6:**
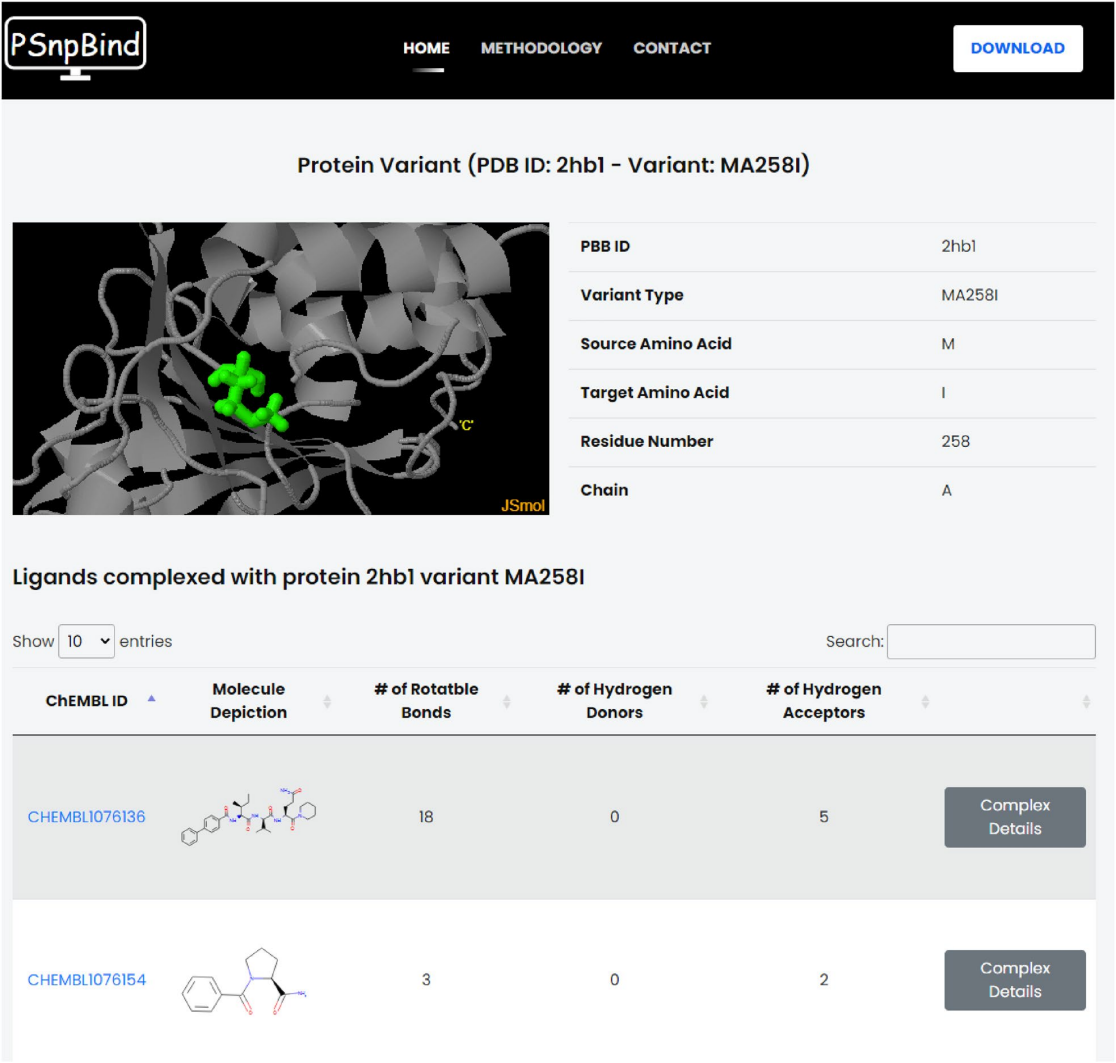
PSnpBind web interface

The PSnpBind front-end (Fig. 6) is implemented using modern web standards and tools (HTML5 [65], CSS3 [66], JQuery^1^ and Bootstrap^2^) and responsive web designs were adopted. Thus, the website can automatically adapt and resize the page layout depending on the screen sizes of a variety of devices. The back-end, allowing communication with the database and handling of the front-end requests was written in Java and used the Spring framework. For the database, MySQL community edition was used to store information about the proteins, mutations, ligands and dockings, and their corresponding folder names in the constructed dataset. The SQL database allows to explore the dataset metadata and links the docking conformers and protein structures files on the disk to the web interface in order to be visualized in the browser. The Jmol molecular visualization library [67] was used to visualize protein-ligand complexes with highlighting the mutations and identifying the close contacts of the drug. The Chemistry Develpment Kit (CDK) [68] library v2.3 is used to generate ligands descriptors. The PSnpBind web application is wrapped in a Docker image, allowing quick and easy deployment on local servers and the cloud. All the code, for both front-end and back-end, is available on GitHub (https://github.com/BiGCAT-UM/psnpbind-webapp).

## Utility and discussion

### Simple search

The search function aims at enabling the user to find or filter the ligands bound to one of the PSnpBind database protein structures based on an input string. The users can search using criteria like CHEMBL ID (of the ligand). String search uses native MySQL regex matching functionality, allowing flexible search in all the columns corresponding to the search criteria. The input string is first wrapped in a regular expression, then the DB is queried against the appropriate fields, and the results, if exists, are sent back to the front-end.

### PSnpBind REST API

A RESTful API is also provided to the users of PSnpBind web application to obtain information about proteins, mutations, ligands and binding complexes (dockings) that are hosted in the database. The API returns JSON objects with a structure corresponding to the entity in question. The API follows the OpenAPI^3^ specification v3, a standardization for how REST APIs are described. A Swagger^4^ UI has been implemented to provide documentation, an interface for users, with little or no programming experience, to ‘talk’ to the services, to quickly and easily formulate queries with the services and obtain dynamically generated source code for popular programming languages, such as Java, Perl, Python and Ruby. The full documentation of the API can be found on (https://psnpbind.org/swagger-ui.html). The PSnpBind supports the following endpoints:

/api/v1/protein/list

/api/v1/protein/PDB_ID

/api/v1/protein/uuid/PROTEIN_UUID

/api/v1/protein/PDB_ID/variants

/api/v1/variant/VARIANT_ID

/api/v1/variant/uuid/VARIANT_UUID

/api/v1/ligand/CHEMBL_ID

/api/v1/ligand/uuid/CHEMBL_UUID

/api/v1/variant/VARIANT_ID/ligand/CHEMBL_ID

/api/v1/docking/VARIANT_LIGAND_UUID

### Molecular visualization

**Fig. 7:**
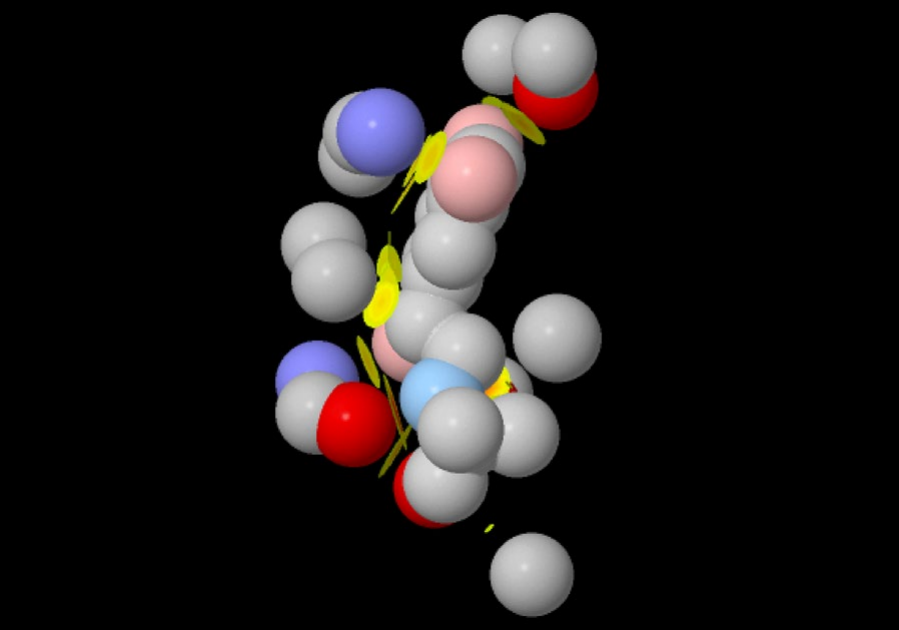
ligand contacts visualization using Jmol. The figure shows the nearest contacts of the ligand. The disks indicate where the van der Waals radii of atoms overlaps. The colors indicate how close the contact is: yellow = close, orange = touching, and red = overlapping

PSnpBind web interface gives users the ability to visualize every protein–ligand docking complex from the constructed dataset using Jmol [67], an open-source Java viewer for chemical structures in 3D. In order to visualize the molecules in the browser, JSmol [69], the HTML5 modality of Jmol, was used which is embeddable in the browser and has all the functionalities of Jmol (the standalone application). The JSmol panel in the web interface (Fig. 7) gives a full visualization experience to explore the protein-ligand complex including: mutation highlight, five different protein representation styles (stick, ball and stick, wireframe, space-filling and cartoon models), molecular surface display and ligand contacts highlights. The nearest contacts of the ligand are visualized using colored disks. The disks indicate where the van der Waals radii of atoms overlaps. The colors indicate how close the contact is: yellow $=$ close, orange $=$ touching, and red $=$ overlapping.

### Structured data (Bioschemas.org) and FAIR implementation

**Fig. 8:**
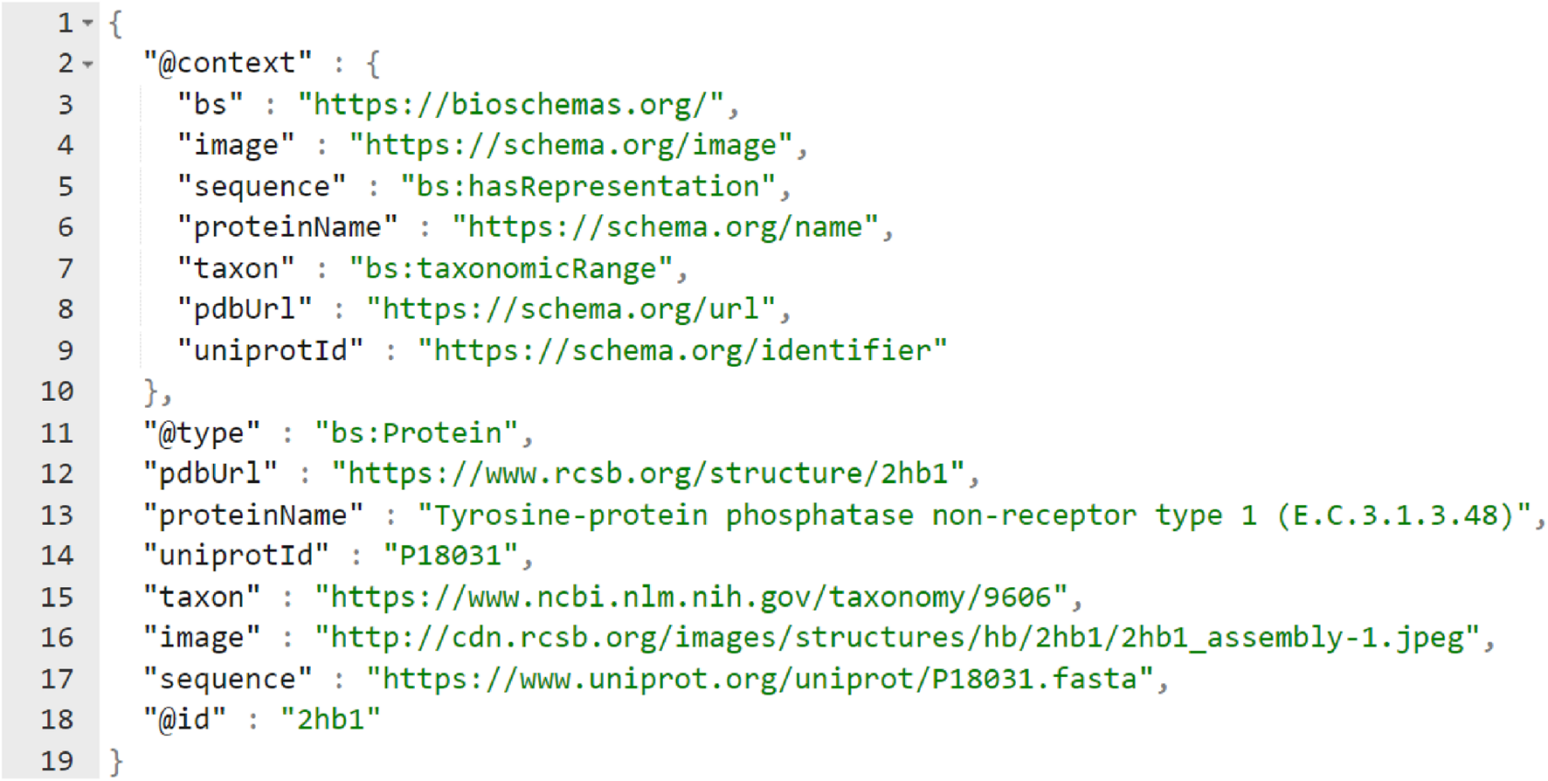
JSON-LD markup example for a PSnpBind protein page, the shemas.org and bioschemas.org vocabularies are used to describe the protein, providing information about the structure, sequence, taxon and IDs

PSnpBind was built with FAIR principles in mind from the ground up. All the FAIR principles were addressed as far as possible. Also, structured markup using JSON-LD is provided for all the pages of proteins and ligands in the web interface. The Bioschemas.org vocabulary [70] was used to annotate the protein information and provide links to PDB, NCBI Taxon and UniProt. Figure 8 shows an example of the JSON-LD generated for one of the protein pages. Also, Table 4 shows a summary of the FAIR principles and their implementation status in PSnpBind.

**Table 4:** Summary of the FAIR principle and their implementation status in PSnpBind

**FAIR principle**	**Implemented**	**Comment**
F1. (Meta)data are assigned a globally unique and persistent identifier	Yes	Internal UUID is generated for each instance of proteins, mutations, ligands and dockings. The database as a whole. the web application, and the libraries made for executing the steps of the workflow are all preserved through Zeonodo with a DOI assigned to each of them.
F2. Data are described with rich metadata	Yes	All instances are annotated and well described from the relevant sources (PDB, UniProt, NCBI Taxon and ChEMBL)
F3. Metadata clearly and explicitly include the identifier of the data they describe	Yes	
F4. (Meta)data are registered or indexed in a searchable resource	Yes	The dataset will be submitted to re3data.org and Google Dataset
A1. (Meta)data are retrievable by their identifier using a standardised communications protocol	Yes	HTTP(S) protocol is used with a REST API for all communications with the server
A2. Metadata are accessible, even when the data are no longer available	Yes	In progress
I1. (Meta)data use a formal, accessible, shared, and broadly applicable language for knowledge representation.	Yes	JSON-LD is used to describe main protein entities. The REST API adopts the OpenAPI specification v3 and it is described using Swagger.
I2. (Meta)data use vocabularies that follow FAIR principles	Yes	The structured markup (JSON-LD) uses the schema.org and bioschema.org vocabularies.
R1. (Meta)data are richly described with a plurality of accurate and relevant attributes	Yes	License, usage and provenance info are all provided.

## Conclusion

PSnpBind is a large database of protein–ligand complexes covering a wide range of binding pocket mutations and a large small molecules’ landscape. This database can be used as a source of data for different types of studies, for example, developing machine learning algorithms to predict protein–ligand affinity or SNPs effect on it which requires an extensive amount of data with a wide coverage of mutation types and small molecules. Also, studies of protein-ligand interactions and conformer orientation changes across different mutated versions of a protein can be established using data from PSnpBind. Furthermore, it provides multiple ways of accessing the data through direct download, a web application and a REST API, all provided in compliance with the FAIR principles. PSnpBind is a valuable resource for new studies in related fields like drug discovery, pharmacogenomics, and structural bioinformatics.

## Data Availability

All PSnpBind data, code to construct the database, and the code for the font- and the back-end of the PSnpBind online website are freely available without any restriction. The obtained dockings dataset can be downloaded from zenodo https://doi.org/10.5281/zenodo.5112334. Code of data processing and database construction workflow is available on GitHub https://github.com/BiGCAT-UM/PSnpBind-Build. Code of the front-end and the back-end is also available on GitHub https://github.com/BiGCAT-UM/psnpbind-webapp.

## Abbreviations

SNP: Single-nucleotide polymorphism
EM: Energy minimzation
API: Application programming interface
CASF: Comparative assessment of scoring functions
SIFTS: Structure integration with function, taxonomy, and sequence
MD: Molecular dynamics
SBDD: Structure-based drug design
SBVS: Structure-based virtual screening
DSRI: Data science research infrastructure
ADT: AutoDock tools
CDK: Chemistry Development Kit

## Footnotes

1 https://jquery.com/.

2 https://getbootstrap.com/.

3 https://www.openapis.org.

4 https://swagger.io/.
